# Role of cognitive parameters in dengue hemorrhagic fever and dengue shock syndrome

**DOI:** 10.1186/1423-0127-20-88

**Published:** 2013-12-05

**Authors:** Jih-Jin Tsai, Kulkanya Chokephaibulkit, Po-Chih Chen, Li-Teh Liu, Hui-Mien Hsiao, Yu-Chih Lo, Guey Chuen Perng

**Affiliations:** 1 Tropical Medicine Center, Kaohsiung Medical University Hospital, Kaohsiung Medical University, Kaohsiung, Taiwan; 2 Division of Infectious Diseases, Department of Internal Medicine, Kaohsiung Medical University Hospital, Kaohsiung, Taiwan; 3 Department of Internal Medicine, School of Medicine, College of Medicine, Kaohsiung Medical University, Kaohsiung, Taiwan; 4 Department of Pediatrics, Faculty of Medicine Siriraj Hospital, Mahidol University, Bangkok, Thailand; 5 Department of Laboratory Medicine, Kaohsiung Medical University Hospital, Kaohsiung, Taiwan; 6 Department of Medical Laboratory Science and Biotechnology, College of Health Sciences, Kaohsiung Medical University, Kaohsiung, Taiwan; 7 Department of Medical Laboratory Science and Biotechnology, College of Medicine and Life Science, Chung-Hwa University of Medical Technology, Tainan, Taiwan; 8 Department of Pathology and Laboratory Medicine, Emory Vaccine Center, Emory University School of Medicine, Atlanta, GA, USA; 9 Institute of Bioinformatics and Biosignal Transduction, College of Bioscience and Biotechnology, National Cheng Kung University, Tainan, Taiwan; 10 Center of Infectious Disease and Signaling Research, National Cheng Kung University, Tainan, Taiwan; 11 Department of Microbiology and Immunology, Medical College, National Cheng Kung University, Tainan, Taiwan

**Keywords:** Flavivirus, Dengue, DHF, DSS, Hemorrhagic, Endotoxin, Fever

## Abstract

Dengue is becoming recognized as one of the most important vector-borne human diseases. It is predominant in tropical and subtropical zones but its geographical distribution is progressively expanding, making it an escalating global health problem of today. Dengue presents with spectrum of clinical manifestations, ranging from asymptomatic, undifferentiated mild fever, dengue fever (DF), to dengue hemorrhagic fever (DHF) with or without shock (DSS), a life-threatening illness characterized by plasma leakage due to increased vascular permeability. Currently, there are no antiviral modalities or vaccines available to treat and prevent dengue. Supportive care with close monitoring is the standard clinical practice. The mechanisms leading to DHF/DSS remains poorly understood. Multiple factors have been attributed to the pathological mechanism, but only a couple of these hypotheses are popular in scientific circles. The current discussion focuses on underappreciated factors, temperature, natural IgM, and endotoxin, which may be critical components playing roles in dengue pathogenesis.

## Introduction

Dengue, a vector-borne human disease, has been recognized recently as one of the most significant public health threats, causing high morbidity and mortality worldwide. The disease is caused by the infection of dengue virus that is transmitted to human beings by the bite of a mosquito– domestic *Aedes aegypti* being the principal vector– although some other species, such as *Aedes albopictus,* are of importance. There are four serotypes (DENV1, DENV2, DENV3, and DENV4), each being capable of inducing typical dengue manifestations. The spectrum of illness is wide, ranging from inapparent or asymptomatic, mild febrile with varying degrees of thrombocytopenia, hemorrhaging and increased vascular permeability typical of dengue hemorrhagic fever (DHF), to plasma leakage and severe shock syndrome. The resurgence of dengue endemicity has resulted from numerous oscillating environmental, social and economical factors. It is estimated that about 40% of the world’s population is at risk of dengue virus infection, with approximately 25 million of these requiring hospitalization and about 25,000 resulting in death [1]. Currently, there are no antiviral modalities or preventive vaccines available to alter disease outcomes. The mortality rate is varying, ranging from 1 to 5%, dependent upon the country and region. The exact mechanism by which dengue virus induces plasma leakage or disease severity remains poorly understood.

A large majority of the dengue infections occur in humans without any noticeable illness. However there are many incidences of symptomatic disease; they can be partitioned into two syndromes: dengue fever (DF) and DHF/dengue shock syndrome (DSS). While DF is a simple, self-limited febrile illness, DHF is a severe and potentially life-threatening condition. DHF/DSS is characterized by thrombocytopenia and hemorrhagic manifestations; additionally, there is increased vascular permeability that leads to depleted intravascular volume and shock. Severe, profound shock, as well as multiorgan failure, is known to occur in extreme cases and is associated with high mortality.

There are many excellent reviews on dengue pathogenesis, including the topics of dengue viral biology, the immune-mediated hypothesis, intervention strategies, and dengue diagnostic issues [2-7]. These aspects will not be included in the focus of the current article; readers who are interested in these details are encouraged to refer to the literature. The current article highlights other recent knowledge and developments in the field, and proposes a new mechanism for biological enhancement to dengue pathogenesis.

### Epidemiology

Initially, dengue disease predominantly affected the people living in tropical and subtropical zones. However the regions of the world that are endemic has spread and the incidence in dengue disease has climbed due to a number of contributing factors. Increased human migration is one culprit; individuals often travel between rural areas and city dwellings and even to other countries via air travel for the purpose of making money or personal enjoyment. A person carrying dengue virus acquired in one location can be bitten again by a mosquito and introduce it into new areas [8]. Another factor is the weather; global warming and climate change has lead to the augmentation of zones hospitable for mosquito survival. Issues with unplanned urban development (including inadequate vector control and poor waste management) have resulted in the presence of many vesicles for the accumulation of water, which are exploited by *Aedes aegypti* for breeding and larvae/pupae production [9,10]*.* All these factors have contributed to the spread of dengue virus in endemic regions.

Recently, dengue has even been spotted in the US territories [11]. In order to avoid a significant impact on the world’s economy and avert potentially extensive burdens to society and the public health sector, a greater amount of research has focused on dengue virus surveillance [12,13]. Consequently, as of today, dengue has been documented in over 100 countries, increasing the number of people at risk for an infection to 2.5 billion people. It is estimated that 50–100 million cases of dengue occur annually, resulting in 250,000 -500,000 cases of dengue hemorrhagic fever (DHF) and 25,000 deaths, depending on epidemic activity. However, these figures are reliant on a number of assumptions and the true incidence is unknown [14-16].

### Diagnosis and clinical presentation

Accurate diagnosis of dengue requires serological testing and identification of viral material in the blood, which is dominantly performed in the clinic. Symptomatology cannot be relied upon because the early symptoms experienced by dengue patients are very similar to most other tropical pathogens and common febrile illnesses. Thus, it is very difficult for attending physicians to attribute the correct pathogen to each clinical presentation when they are often highly variable. Once the physicians determine the differential diagnosis, the second layer of difficulty is to distinguish whether the patient has dengue fever or dengue hemorrhagic fever. The former is likely a self-limited illness and patients normally recover without having noticeable sequelae; in contrast, the latter, if treatment is not instituted immediately, the progression of the condition can quickly escalate and result in life-threatening situations, including death. According to the old WHO guidelines [17], the initial phase of clinical manifestations for DF and DHF were quite similar. In general, the onset of DF and DHF are both very abrupt, beginning with fever. The common initial symptoms at the febrile stage are headache, malaise, weakness, chills, aches and pains, and gastrointestinal symptoms. Physical examination often reveals flushing of the face, lethargy, irritability (in young children), abdominal pain, hepatomegaly, and the presence of petechial hemorrhages or other bleeding manifestations. Initial complete blood counts reveal leucopenia, and after 2–5 days of fever, thrombocytopenia and depletion of coagulation factors often develop.

In DF, the fever abates after 3–7 days and the patients recover. In DHF, signs of progressive intravascular fluid leakage, such as petechiae, ecchymosis, epistaxis, gingival or gastrointestinal bleeding, occur after 3–5 days of fever. Frequently, confluent petechial convalescent rashes with scattered sparing spots develop in patients that undergo plasma leakage, allowing doctors a way to differentiate between DHF and typical DF. Conditions arising from plasma leakage, including pleural effusion, ascites, and hypoproteinaemia, are common in severe dengue. This is the so-called critical stage, when the fever typically begins to dissipate but the patients’ condition may worsen. At this point many patients may develop shock from depletion of intravascular volume and bleeding. Some patients deteriorate rapidly from circulatory failure, experiencing a condition called dengue shock syndrome (DSS), presenting with a rapid and weak pulse, narrow pulse pressure or hypotension, cold clammy skin, and altered mental status. Disease severity is classified as either mild (grades I and II) or severe (grades III and IV), the presence of shock being the main difference. This stage lasts no more than 48 hours, after which the patients usually recover [16].

This WHO classification has been mostly adequate and used for many decades; however there have been occasional difficulties in classifying patients who present with unusual manifestations. Atypical or abnormal clinical presentations have been reported such as encephalopathy, severe hepatitis, and myocarditis, in which the patients have severe disease but do not fit the DHF definition. In 2009, WHO published another case classification system for guiding dengue management [16]. This new classification includes dengue without warning signs, dengue with warning signs, and severe dengue, which improved sensitivity for detection but reduced specificity [18,19]. To improve upon the specificity of the 2009 dengue classification system, in 2011 WHO SEARO published an amendment, which expanded case definitions based on the previous DF/DHF (WHO 1997 [17]) description to include unusual manifestations [20]. Both WHO guidelines, the 2009 and SEARO 2011 versions, are in use in several countries. However, this is dependent upon the country’s public health administrative leaders; some advocate classifying disease according to the new guidelines, while others still triage patients according to the expanded older classification of DF and DHF.

Due to the nonspecificity and complexity of dengue patient clinical manifestations, it is imperative to confirm the clinical diagnosis with biological and/or laboratory assays. Since dengue disease management is time sensitive, onsite rapid screening tests at the point-of-care is a critical component in assisting decision-making. Unfortunately these rapid screening tests perform poorly, having low specificities and sensitivities. Other diagnostic tools, such as virus isolation or dengue virus genome, antigen or specific IgM/IgG detection, are more informative but are also very time consuming and expensive to perform. Even though these results do not directly contribute to decisions for the patients in real time, the confirmatory information can provide a guideline for future decision making on patient care in general, as well as lead to improved precision on diagnostic rapid screening tests under development. In addition, the results obtained with confirmatory assays can serve to advance our understanding of human dengue virus pathogenesis and guide the development of preventive modalities.

### Parameters associated with DHF/DSS

Although the majority of dengue-infected individuals are asymptomatic, a small percentage of the subjects will progress to apparent clinical illness including life-threatening DHF/DSS. The available information suggests that multiple factors, including the presence of cross-reactive, sub- or non-neutralizing antibodies, viral virulence, genetic predisposition, age, nutritional status and underlying chronic disease, can all be a risk and/or contributive factor to the pathogenesis of DHF/DSS [21-23]. However none of these factors has been substantiated because there are no reliable in vivo model systems to perform the necessary side-by-side comparisons. Consequently, the causes of dengue disease remain poorly understood in spite of many decades of intensive investigations. Some known but under-appreciated factors are briefly discussed here.

#### Fever temperature

Temperature has captured the attention of the media, owing to growing concerns about the environment and global warming. In line with this, the change in theglobal climate has significantly impacted the geographic distribution of the mosquito vector and thus dengue disease. Accordingly, WHO has reported that a temperature rise of only 1–2°C could increase the risk of dengue virus infection to the population by several hundred million, potentially resulting in 20,000–30,000 more fatal cases annually [24]. Additionally, the biological importance of temperature, particularly in the form of fever, and its role in medical science has not received the appropriate attention [25]. Dengue fever, as the name indicates, has fever as one of its most salient clinical features. This is also the case for many other common febrile illnesses. The increase in body temperature during infection is commonly viewed of as one way to interfere with pathogen replication directly. Additionally this biological alteration may also promote the production of the appropriate host transcriptional and translational profiles, which may work to eradicate some microorganisms. Despite these known phenomena, the contribution of fever to pathogenesis has not been investigated. Researchers more frequently attribute the symptoms as directly or indirectly caused by the pathogen rather than a direct result of the fever. Refocusing the interpretation of the clinical data in light of the degree of fever may allow for a better understanding of disease presentation.

**Figure 1:**
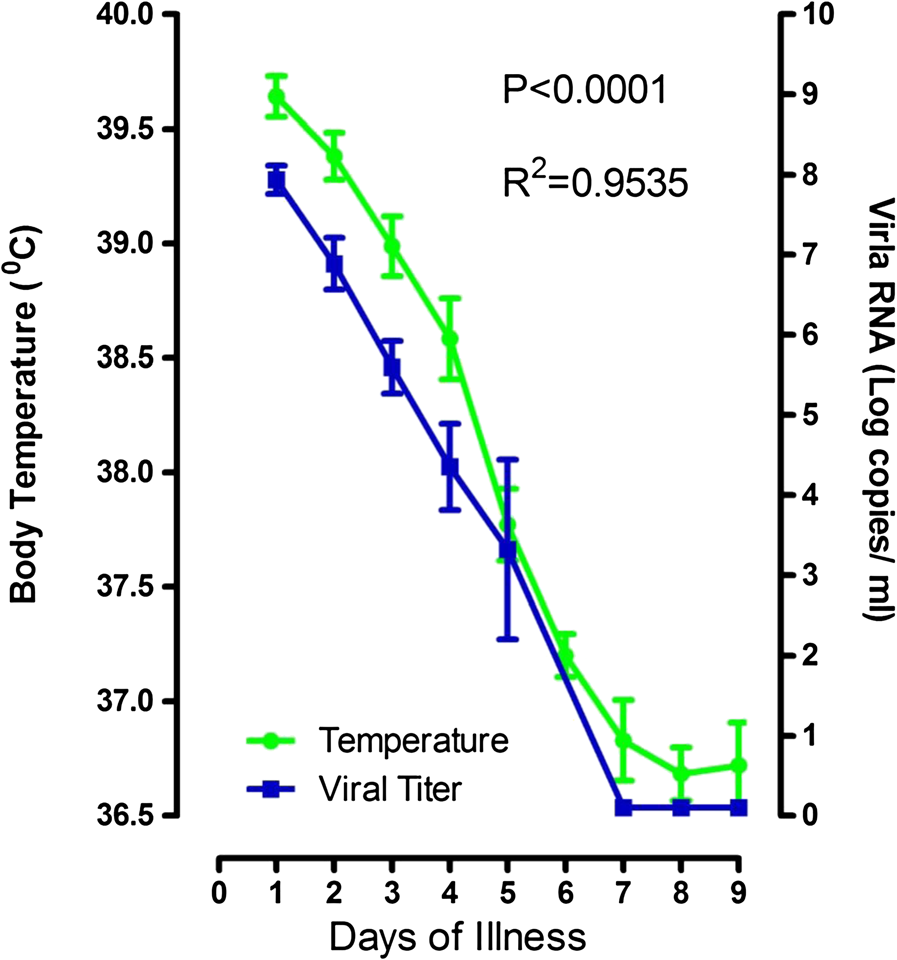
**Association of body temperature and viral load.** Body temperature and samples were measured from 147 walk-in patients. Viral load was quantified by real-time RT-PCR as described elsewhere [31]. Both body temperature and viral load decrease with time.

Viremia is another major finding on the pathophysiology of dengue patients. Interestingly, it has been noticed that viremia is correlated highly with temperature in dengue patients [26]. A cumulative result from a pilot study supports this observation as well (Figure 1). Viremia kinetics are characterized by a downward trend, with a peak in the plasma viral RNA levels corresponding to the first day after onset of fever, which decreases to undetectable levels by the 7th day of fever. The downward trends in viral RNA level and body temperature are very similar to each other and are correlated highly with each other as a function of time (P < 0.000, R^2^ = 0.9535). The results were in line with reports on the highly correlation of body temperature with viral load in samples collected from acute dengue patients [26]. One interpretation of this data would suggest that the lower body temperature contributes to the clearance of viremia. However the results could also suggest that dengue virus may enter cells or replicate more efficiently at higher temperatures [27,28]. Interestingly, it has been reported that Flaviviruses in *Aedes albopictus* cell cultures adapted to 34.5°C replicate to a higher viral titer than those adapted to 28°C [29] and that Japanese encephalitis virus yields are increased by 0.2-2.5 log PFU/ml in heat shock-treated BHK-21 cultures at 41°C compared to control cultures at 37°C [30]. However, why viral titers are amplified when culturing at higher temperatures remains to be further investigated. Foreseeably, understanding the factors or mechanisms leading to efficient viral replication during fever, would provide a new avenue of strategies to improve the quality of life of affected patients and perhaps a preventive modality to dengue as well.

#### Biological enhancement

**Figure 2:**
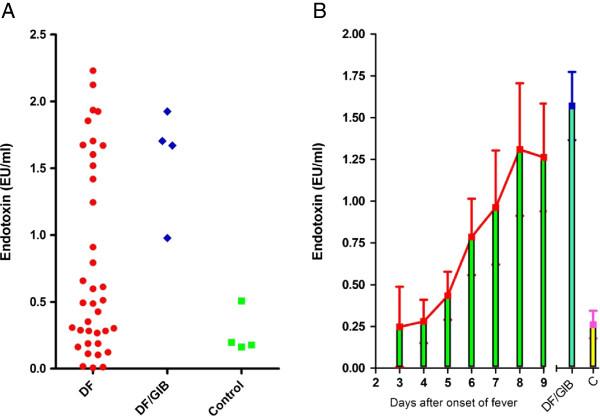
**Endotoxin observed in sera of dengue patients. (A)** The levels of endotoxin in 37 randomly chosen samples were measured by ToxinSensor^TM^ Chromogenic LAL Endotoxin Assay Kit (GenScript USA Inc., Piscataway, NJ). Two distinct patterns were observed in DF patients. A high range of endotoxin levels were observed in a fraction of DF patients and in DF patients with noticeable gut bleeding, while a subset of DF patients had endotoxin levels within the range of healthy controls. **(B)** The levels of endotoxin in general, increased with time, being higher on days 6 to 9 after onset of fever.

Pathophysiologic responses to dengue virus infection are dynamic. Biological components circulating in the patient’s blood stream may modify the body’s physiology and alter the presentation of the disease. The biological response to the disease is a double-edged sword; it could be both beneficial and harmful to the host. Shock syndrome often occurs during or at the end of the viral clearance stage and is a dangerous complication of dengue virus infection that is associated with a high mortality rate in some countries [4]. Increased vascular permeability is one of the remarkable clinical manifestations that have been observed in patients with severe dengue. This event may provide a mechanism for the translocation of microbial products from the intestinal lumen into the circulatory system [32,33]. Although sepsis can be observed clinically in patients experiencing DSS and those with bacteremia [34], the mechanism leading to the development of dengue shock is complex and remains largely unknown. Interestingly, gut injury has been correlated significantly with multiorgan failure in hemorrhagic shock [35]. The degree of this mucosal injury in dengue patients has been reported to correlate with the severity of the illness [36]. Recent reports indicate that more than 35% of DF patients have evidence of bleeding in the gut as well [37]. Also, there appears to be an escalating problem with opportunistic pathogen infections in dengue patients (Table 1). This suggests that substances in the gut may translocate and become systemic. One of these such materials is lipopolysaccharide (LPS) or endotoxin [38], a strong immune response inducer. Interestingly, it has been reported that endotoxin is detected in 50% of serum samples collected from DHF/DSS patients [39], and that the levels of LPS in these samples correlate with dengue severity [40]. In a study with cumulative data, we observed that endotoxin levels were significantly higher in dengue patients confirmed with gastrointestinal bleeding (GIB) and that 48.6% of sera from acute DF patients were considered to be endotoxin positive compared to healthy controls (Figure 2A). Kinetic studies demonstrate that the highest levels of endotoxin were seen at the end of disease from days 6 to 9, during defervescence (Figure 2B); this time point also corresponds with the critical stage, during which patients need to be closely monitored for the occurrence of shock. The sera from the GIB group were sampled from the 4th day after the onset of fever. However, although results were derived from limited patients and the actual percentage of GIB remains unknown, literature reports have estimated that the percentage of the GIB has been varied from regions to regions, ranging from 1 to 39 percent [37,41,42]. Therefore, clinically, GIB is not only largely unknown but also underestimated because there may be some occult bleeding that were overlooked and asymptomatic presentation as well. The demographic and clinical data of the enrolled patients have been previously described [43,44].

**Table 1:** Escalating problems of opportunistic pathogen infections in dengue patients

**Cases**	***Pathogens***	**Day of fever**	**Reference**
5	*Staphylococcus aureus*	8-10	[45]
4	*Staphylococcus aureus, Haemophilus influenzae, Coagulase-negative staphylococcus*	7-10	[46]
14	*Burkholderia pseudomallei, Varicella zoster, Salmonella, Shigella, Escherichia coli, Herpes simplex, Mycobacterium tuberculosis, Streptococcus pneumoniae, Mycoplasma pneumoniae*	7-10	[47]
2	*Salmonella typhi*	8	[48]
1	*Shigella sonnei*	9	[49]
7	*Rosemonas species, Klebisella pneumoniae, Moraxella lacunata, Klebisella ozaenae, Enterococcus faecalis*	8-14	[50]
3	*Enterococcus faecalis, Klebisella pneumoniae*	unspecified	[51]
4	*Aspergillus fumigatus*	8-13	[52]
5	*Plasmodium vivax, Plasmodium falciparum*	3-10	[53-56]
2	*Leptospira*	5-7	[57,58]
1	*Candida tropicalis*	14	[59]

**Figure 3:**
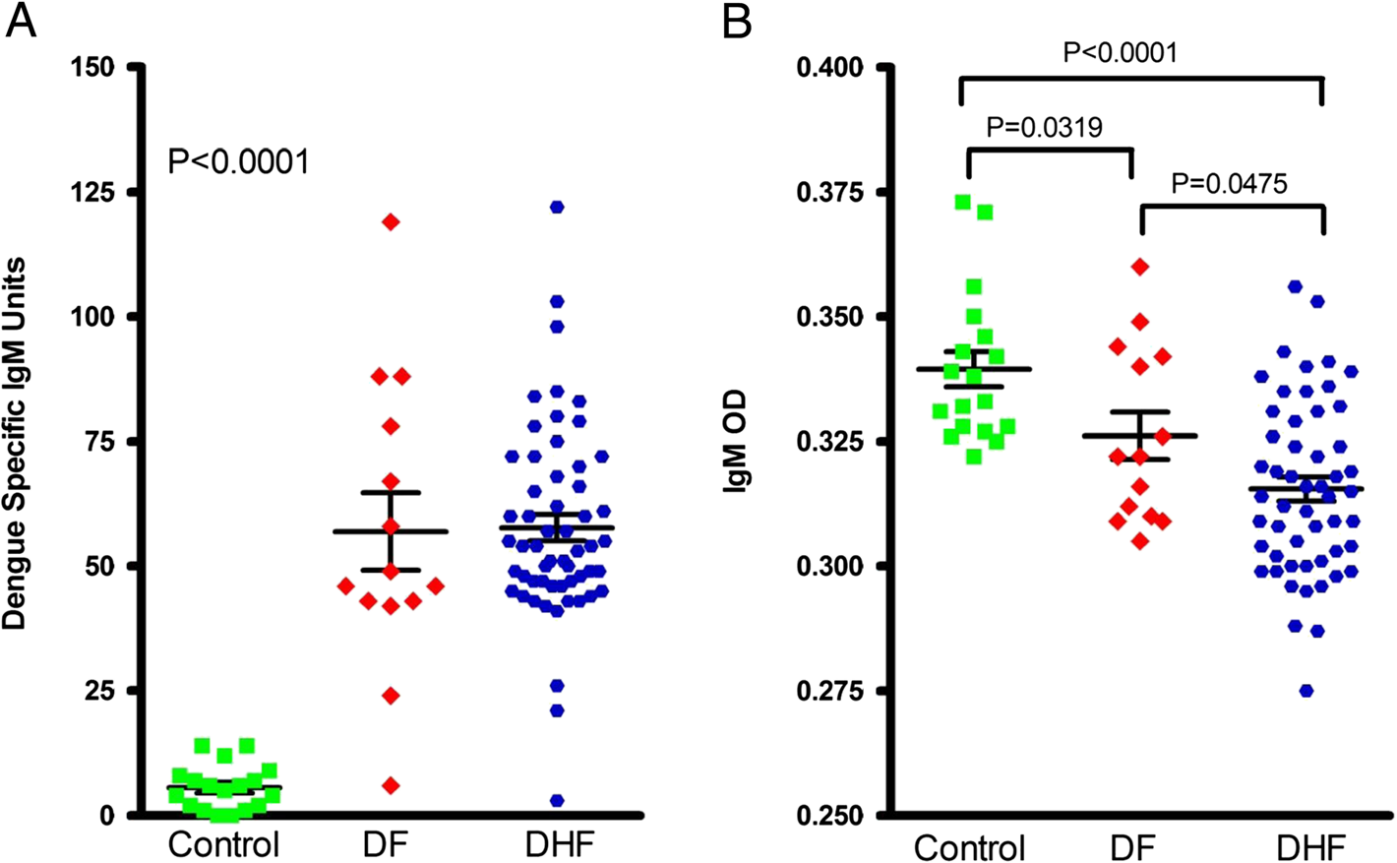
**Natural IgM was significantly lower in dengue patients. (A)** Dengue specific IgM was measured as previously described [71]. Dengue specific IgM was only present in dengue patients. **(B)** Total IgM was measured as previously described [72]. The levels of total IgM were significantly lower in both DF and DHF patients compared to healthy subjects.

One mechanism known to rid the blood stream of endotoxin is through antibodies or immune complex formation. Serum antibodies are a heterogeneous mixture of immunoglobulins (Ig), all of which share the ability to bind individually to specific antigens. In mammals there are five classes of antibody: IgA, IgD, IgE, IgG and IgM, with 4 IgG and 2 IgA subclasses present in humans. The IgM antibody is the first class of antibodies produced during a primary response. Studies from mice reared germfree and receiving an antigen-free diet have the same serum IgM levels as mice held under conventional housing conditions, while IgG and IgA levels in these mice are greatly reduced, suggesting that a distinct antibody, natural IgM, is induced independently of external stimulation [60]. Later, it was shown that IgM is the major component of natural antibody in humans [61,62]. The levels of natural antibodies vary among individuals, but increase with age and with good nutrition [63-65]. Characteristics of the natural IgM antibody include low affinities and broad specificities to both foreign and self antigens [66]. Circulating natural IgM antibody provides the first line of defense against invasion by pathogens [67-69]. Importantly, natural IgM has been demonstrated to play an important role in the clearance of endotoxin [70]. Pilot results from sequential samples suggested that the levels of total IgM are dose dependent with disease severity; DHF patients have significantly lower levels of total IgM in sera than DF patients, in spite of similar levels of dengue specific IgM; and the total IgM from the sera of both DF and DHF were significantly lower than that of healthy controls (Figure 3). The results imply that dengue patients typically have lower IgM than that of healthy controls and possible a reduced capability to clear LPS, lending support to the hypothesis that endotoxin may contribute to disease pathology.

Natural IgM is high avidity of polymeric antibody, which may contribute to the initial immune defense and to the control of invading pathogens until immune system has time to launch a specific adaptive response [73]. Importantly, natural IgM antibody has been shown to directly neutralize or inhibit pathogens as well as aid the initiation of adaptive immune response from follicular B cells, which together play critical roles in protection against bacterial and viral infection [67,69,74-76]. Consequently, despite with limited number of specimens, we feel confident that the levels of the IgM in acute dengue patients could be lower than that of healthy subjects. Interestingly, recent evidence also suggests that lipopolysaccharide levels are elevated in dengue virus infected patients and correlate with disease severity [40].

One of the alternative contributing factors is the amount of platelets. Dysfunctional platelets and thrombocytopenia are a salient clinical finding in dengue patients and are correlated with the severity of disease [77]. A platelet-endotoxin interaction is a necessary step for the final removal of LPS by the reticuloendothelial system [78]. The evidence also suggests that the levels of detectable endotoxin in patients may be inversely correlated with the platelet counts. Some percentage of dengue shock cases may result from increased gut mucosa permeability, which could lead to abnormally high endotoxin levels in the peripheral blood. This phenomenon in combination with reduced platelet counts and reduced IgM specific to LPS could lead to inefficient clearance of endotoxin and consequently another mechanism will need to be induced to promote its removal from the bloodstream.

Scientifically, it has been known that phagocytic cells such as primary monocytes and macrophages are very difficult to get infected by dengue virus [79]. But, if these cells are pretreated with endotoxin (LPS) [80], the infectivity rate increases significantly, likely as a result of enhanced phagocytic activity [72]. Monocytes potentially acquire the virus when they engulf dengue-containing platelets, a frequent occurrence in dengue patients on days 6–8 after the onset of fever [81,82]. In addition, LPS is known to bind to the CD14 receptor of macrophages and B cells and promote the secretion of pro-inflammatory cytokines [83,84]. Interestingly, it has been suggested that activated macrophages from secondary DENV infected patients display enhanced phagocytic behavior of opsonized platelets, through a mechanism involving milk fat globule-epidermal growth factor 8 [85]. Taken together, a hypothetical scenario can be drawn; endotoxin, usually kept at a low frequency in the circulation by functioning platelets, may leak into the periphery through a damaged gut-endothelial barrier in dengue patients, whom likely have dysfunctional platelets, thrombocytopenia, or low natural IgM and are unable to clear off the endotoxin in a timely manner. This combination of events may result in the induction of activated macrophages or monocytes, enhancing their engulfment activities and triggering a tsunami of inflammatory cytokine production and inciting septic shock. However this alternative hypothesis requires further investigation.

#### Pre-existing immunity

The pathophysiology of severe dengue is very complex and may involve multiple factors. Epidemiological data tabulated from dengue endemic locales suggest that serologically defined primary dengue virus infection and/or subsequent homologous serotype infection is known to be associated with less severe disease as compared with secondary subsequent heterologous serotype infection, a term has been coined as antibody dependent enhancement [86]*.* However, our understanding of these interacting components that contribute to the development of dengue disease is obstructed by the lack of suitable animal models that can recapitulate the cardinal features of human dengue. As a result, the exact mechanism(s) leading to the development of DHF/DSS remains poorly understood, in spite of several decades of intensive investigations. One of the factors believed to play a role in pathogenesis is pre-exposure. Results available from dengue epidemic countries have indicated that severe disease more frequently occurs not with the primary but during subsequent viral infections [87,88]. Without experimentation with the appropriate comparison groups and controls, it became assumed that pre-existing immunity following a challenge with a heterogeneous serotype is a risk for DHF/DSS. Consequently, the hypothesis suggests that DHF/DSS results from an abnormal or exaggerated host immune response -– particularly due to the cross-reactive antibodies, which bind similar epitopes on other dengue viral strains – that augments the rate of virus uptake [4,71,89]. However, recent results accumulated from non-dengue endemic regions [90] and from travelers suggest that the frequency of DHF in primary infections in naive individuals is similar to that of secondary infection [91]. Also, Libraty et al’s cohort study reveals no association between maternal antibodies and development of severe dengue in infants [92]. Collectively, multiple causes may play a critical role in dengue pathogenesis. The cause of pathology in naïve individuals and in infants infected by dengue virus may be distinctively distinguishable from that of primary and secondary infection, respectively, in dengue epidemic zones.

**Figure 4:**
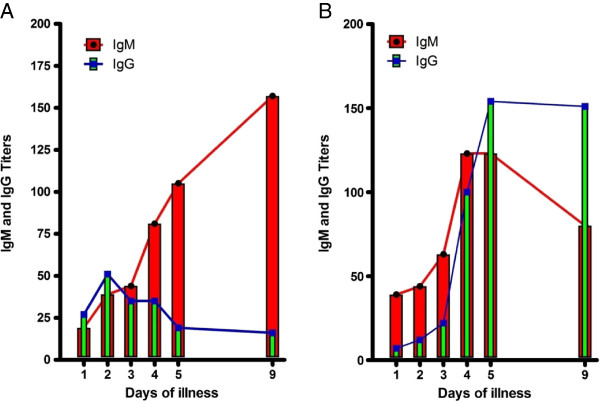
**Abnormal antibody response observed in sequential samples in dengue patients.** Sera were collected at the indicated days and the titers of antibodies were measured as previously described [71]. **(A)** Primary dengue virus infection. These samples were determined to be from secondary patients by the IgM/IgG ratio recorded at the early acute stage. There is no robust amplification in IgG, indicating that these patients should be recategorized as primary infections. **(B)** Secondary dengue virus infection. These samples were determined to be from primary dengue patients by the IgM/IgG ratio recorded at the early acute stage. These samples demonstrate IgG amplification, indicating that these patients should be considered secondary patients. IgM/IgG ratios, obtained at the acute stage, cannot accurately distinguish between primary and secondary virus exposure in dengue patients.

According to the WHO guidelines, it is required that several specimens within a certain time interval be processed to clearly define the infection as primary or secondary. But, very often, in the clinical setting, multiple sample collection is inconvenient or dangerous to collect. Thus the term primary and secondary in dengue epidemic zones are often defined with a single collection sample by the ratio of IgM/IgG; if the value is >1.2, then it is a primary infection, but if the value is ≤1.2, it is a secondary. However, a very high dengue antibody prevalence rate of 85-95% is seen in school-aged children in epidemic countries [88,93,94]. Also, IgG is characteristically unusually low at the onset of disease in secondary dengue patients [95]. Consequently, the definition cannot distinguish between current infection and previous infection. Frequently it is furthermore complicated by samples with similar (at 1) or slightly below 1.2 ratios for IgM and IgG. This case is very often arbitrarily assigned to be secondary infection, and thus the definition has been called into question [96]. To test whether this practice can accurately distinguish between primary and secondary sequential samples were obtained from a cohort study. Sera were collected daily for 7 days from 30 confirmed dengue patients. The IgM/IgG ratio was measured after antibody titers were determined. This study indicated that if the early time point samples were used to define the primary and secondary, then the wrong category was often assigned since the IgM/IgG ratios at the later time samples clearly suggested that the allocation should be to the opposite category (Figure 4). Although the actual percentage of the erroneous allocation of the category is unknown, primary and Secondary dengue assignments based on the >1.2 IgM/IgG ratio may intricate the pathogenic cause of dengue in endemic countries. However, we observed about 26.7% (8/30) abnormal antibody response.in current investigation, the percentage therefore seemed to be underestimated in dengue epidemic zones. Thus, a better test that can differentiate primary from secondary dengue virus infection is urgently needed.

As a whole, evidence for the role of pre-existing immunity in human disease is still by and large circumstantial [23,97,98]. Thus, in order to further advance the understanding of the causes of DHF/DSS, reported disease should be divided into three major categories (naïve primary infection, defined primary infection in endemic zones, and secondary infection) and considered separately [99]. With a clearer definition of the virus pre-exposure history, the search for the identity of the pathogenic cause for DHF/DSS may be much simpler to assess and faster to acquire and likely make much more sense.

#### Viral strains

The occurrence of DHF/DSS in primary naïve individuals and the high frequency of asymptomatic secondary infections implicates that the immune-enhancement hypothesis alone is inadequate to explain dengue pathogenesis. An alternative explanation for the pathogenesis of DHF/DSS is the virulence of different viral strains [100]. Although the in vivo scientific data on the topic is quite sparse, it can be interpreted that some dengue viral strains are more virulent for man than others. Reports based upon the epidemiological data advocate that particular serotypes appear to be more virulent than others with certain ethnic groups [101-106]. In addition, experimental results also suggest that certain genotypes within a serotype encode determinants for virulence, attenuation, and tissue tropism [107-111]. However, substantiation of the virulent strain hypothesis of dengue pathogenesis still awaits the availability of an adequate disease model for validation [112,113].

#### Other factors

As aforementioned, the factors that place patients at higher risk of developing DHF/DSS are not clearly identified yet. Multiple factors have been correlated with DHF/DSS: age, sex, underlying disease, nutritional status, ordering of serotype pre-exposure, individual genetic background including HLA type and ethnic variation [114-119]. These factors have yet to be further evaluated.

### Treatment and prevention

Currently, there is not a specific antiviral treatment for dengue. Even if there were a drug available that could reduce viral replication or entry it would have limited usage. Treatment of dengue disease is time-sensitive; in other words, as time progresses, the presence of the virus and the ability to accurately detect it decreases, while the risk of severe immune-mediated disease increases. The best treatment currently available is immediate supportive or palliative care with vigilant monitoring by the professional healthcare staff. Patients usually recover after fluid and electrolyte supportive therapy. Early recognition of DHF and immediate treatment are of utmost importance to reduce the case fatality rate.

Since there is no antiviral therapeutic modality or vaccine against dengue available, the only possible preventive method that can be instituted is mosquito control. However, the effectiveness of current insecticides is diminishing and the successfulness of this strategy is compromised by its high cost. Thus, a dengue vaccine is urgently needed to prevent the virus from further spreading.

## Review; Conclusion

Dengue has been associated with human beings for more than two centuries and yet its pathogenic cause(s) remain poorly defined. Lack of a suitable animal model recapitulating the cardinal features of human dengue further hinders the progress of our understanding. Numerous factors and hypotheses have been associated with or attributed to the pathogenesis of dengue. There are only limited results suggestive that some of these theories may be the primal instigator of severe disease; however they remain to be circumstantial and require further verification. Complexity of severe dengue suggests that other factors, such as fever and endotoxin, are important as well. These factors are often underappreciated and may not only provide the critical link in understanding the cause(s) of dengue pathogenesis, but offer a new strategy for the amelioration and/or prevention of dengue.

## Competing interests

The authors declare that they have no competing interests.

## Authors’ contributions

JJT desingend and drafted the IRB protocol and enrolled the patients, KC drafted the IRB protocol and enrolled the patinets and edited the manuscript, PCC enrolled the patients and collected the samples for the studies, LTL assisted in performing the laboratory assays and analyzing results, HMH performed the laboratory assays, YCL assisted in data analysis and edited the manuscript, and GCP wrote and edited the manuscript. All authors read and approved the final manuscript.
